# Shaping swine career identity through Pork Prospect: a mixed methods study in Minnesota and Ohio

**DOI:** 10.3389/fvets.2026.1927703

**Published:** 2026-10-02

**Authors:** Samira Chatila, Kara Flaherty, Pedro E. Urriola, Maria Pieters, Jacqueline Nolting, Talita Pilar Resende

**Affiliations:** 1 Department of Animal Science, University of Minnesota, Minneapolis, MN, United States; 2 Department of Veterinary Preventative Medicine, The Ohio State University, Colombus, OH, United States; 3 Department of Veterinary Population Medicine, University of Minnesota, Minneapolis, MN, United States; 4 Department of Veterinary Diagnostic Laboratory, University of Minnesota, Minneapolis, MN, United States

**Keywords:** career development, experiential learning, food animal, mixed methods, swine careers, veterinary education

## Abstract

**Introduction:**

This study examined Pork Prospect, a three-component swine-focused experiential learning program designed to support food-animal veterinary workforce development by scaffolding learners’ career exploration, occupational fit appraisal, and professional identity formation. The program was evaluated using the PIG Pathway Framework, which integrates Possible Selves Theory, Social Cognitive Career Theory, and Experiential Learning Theory across three developmental stages: Exploratory (high school), Formative (college), and Immersive (early career).

**Methods:**

Using a qualitatively driven mixed methods design, we evaluated program outcomes among 167 participants in Minnesota and Ohio, including high school students (*n* = 91), college students (*n* = 58), and advanced undergraduates or early-career professionals, including graduate students and recent graduates (*n* = 18). High school and college participants attended a one-day “Swine Discovery Day” consisting of educational presentations and a visit to a swine research facility. Early-career participants completed a “Swine Bootcamp,” consisting of individualized, professionally mentored experiences lasting 2 days to 2 weeks and tailored to participants’ career interests in swine production, veterinary medicine, research, extension, and other industry settings. Pre- and post-program Likert-scale surveys were analyzed using Wilcoxon signed-rank tests for high school and college groups. Post-program survey data and open-ended responses were analyzed descriptively and thematically.

**Results:**

High school participants showed significant gains in comfort around pigs, swine industry knowledge, and career awareness, suggesting that early exposure expanded possible career selves. College participants demonstrated significant increases in industry knowledge, career awareness, and consideration of swine-related careers, indicating that structured experiential contact supported appraisal of occupational fit, perceived barriers, and career possibilities. Early-career participants reported strong satisfaction, perceived knowledge gains, and greater clarity and confidence regarding swine-focused career pathways, suggesting that mentored immersion supported professional rehearsal and identity consolidation. Across program components, qualitative themes highlighted the importance of hands-on learning, mentorship, reflection, and embodied encounters with swine work in shaping motivation and professional commitment.

**Discussion:**

Findings suggest that developmentally sequenced experiential programs may support food-animal career development by matching learners’ educational stage with appropriate forms of exposure, reflection, mentorship, and immersion. The study contributes a theory-informed model for understanding food-animal veterinary career development as a staged process of professional becoming rather than a linear pipeline from exposure to career choice.

## 1 Introduction

Animal agriculture in the United States (U. S.) faces a persistent workforce challenge, including the decline of domestically trained food-animal professionals and broader concerns about the stability of the agricultural labor force. Veterinary medicine and animal science programs, key training grounds for future food-animal specialists, are struggling to attract and retain students into livestock-related careers. Despite a significant increase in the number of animals raised for food, the U. S. has experienced a dramatic decline in food-animal veterinarians (FAVs), losing nearly 90% of its large-animal practitioners since World War II. Reported barriers to recruitment and retention include student debt, fewer rural placement opportunities, lower compensation compared to companion animal practice, mental health issues, and fear of being discriminated against on the basis of gender, race, and other aspects of their identity (1–4).

Against this backdrop, the swine industry offers a useful case for examining food-animal recruitment and retention. As one of the largest sectors of the U. S. animal agriculture, the swine industry is often underrepresented in veterinary curricula (5) and student interest in this area remains low (4). Taken together, these characteristics underscore the need for educational models that cultivate and retain talent, while attending to how students encounter the industry across different stages of their educational development. This workforce challenge is also important to the veterinary profession in particular because food-animal veterinarians support herd health, disease prevention and surveillance, biosecurity, animal welfare, food safety and security, and access to veterinary services in rural communities (2, 4). The limited representation of swine medicine in veterinary curricula may also restrict students’ opportunities to understand this work before making specialization decisions (5). Veterinary education therefore needs developmentally appropriate opportunities that introduce learners to food-animal practice, help them realistically evaluate occupational fit, and connect interested students with mentors and professional networks as early as possible.

In response, this study examined Pork Prospect, a three-component swine-focused experiential learning program: Swine Discovery Day-Youth (SDD-Y), an exploratory component for high school students; Swine Discovery Day-College (SDD-C), a formative component for college students; and Swine Bootcamp, an immersive component for advanced students and early-career participants. To guide the design and sequencing of the components, the PIG Pathway Framework was developed (Table 1), which aligns experiential learning with the evolving needs, identities, and motivations of learners across educational stages. The framework scaffolds experiences from curiosity and exploration to commitment and career clarity. The study addresses the following research question: How does Pork Prospect influence participants at the levels of career exploration, identity development, and professional commitment in the swine industry?

**Table 1:** The PIG pathway framework is a developmental model that integrates possible selves theory (P), identity, motivation, and self-regulation constructs from social cognitive career theory (I), and grounded experience (as per experiential learning theory) (G) across three stages of learner progression: exploratory, formative, and immersive.

Developmental stage and purpose			PIG pathway		
Learner developmental stage across pork prospect program components	Stage description	Primary developmental goal	(P) Possible selves	(I) Identity, motivation, and self-regulation	(G) Grounded experience
Exploratory (SDD-Youth)	Youth considering a range of future selves and may be (un)familiar with swine careers.	Spark early curiosity by making swine careers visible and approachable.	Forming “future self” in agricultural and veterinary pathways; expanding perceived possibilities.	Emerging self-efficacy shaped by encouragement, role models, and early affective responses.	Initial hands-on exposure to swine and farm settings activates engagement.
Formative (SDD-College)	Students committed to animal/vet fields but still clarifying career direction.	Support reevaluation of interests in food-animal careers with a focus on swine.	Reevaluating one’s possible selves based on academic and field experiences.	Domain-specific self-efficacy develops; students assess barriers, supports, and outcome expectations.	Reflection deepens; students begin aligning values and identity with specific swine roles.
Immersive (Swine Bootcamp)	Learners with strong interest seeking confirmation and clarity.	Facilitate transition from interest to professional intent through immersive experience.	Consolidating a professional future self that is aligned with real-world career goals.	High self-efficacy and motivation interact with structural factors to shape career planning.	Full experiential cycle: immersion, reflection, application, and goal-setting in professional contexts.

The full framework combines learner characteristics, developmental goals, and theoretical mechanisms to guide experiential career programming. The grey-shaded boxes highlight the PIG Pathway component that plays the most conceptually central role at each developmental stage.

## 2 Theoretical framework

National programs that serve youth, such as 4-H, National FFA Organization, and similar initiatives, have long played a vital role in introducing students to agricultural careers and helping develop early interest, leadership skills, and a sense of belonging within relevant communities (6–8). However, upon entering higher education, students encounter far fewer programs that provide intentional, species-specific scaffolding or structured opportunities to explore and evaluate swine-focused career pathways. As a result, the support available to help make informed decisions about specialization becomes markedly narrower. For example, many first-year veterinary students at the University of Minnesota reported limited or no experience with pigs and had not previously visited swine farms (9).

Several educational interventions have attempted to spark interest in food animal veterinary careers through lectures, farm visits, and short-term youth programs (9–12). Other more structured and longer-term educational interventions include elective undergraduate courses that introduce students to veterinary careers and mandatory professional development courses for veterinary students that integrate lectures with on-farm visits (9, 10). However, such opportunities remain unevenly distributed across universities, highlighting the need for shorter-term, scalable interventions that recognize how learners at different stages benefit from distinct forms of engagement.

Yet, much of the literature on food-animal career development treats exposure as a relatively straightforward mechanism whereby students are presumed to become more interested in food-animal careers once they gain information, contact, or experience. This assumption risks flattening the social and developmental complexity of career formation. Learners do not merely acquire knowledge about swine careers; they also assess whether such careers are imaginable, desirable, socially supported, and compatible with their emerging identities and life expectations. In this sense, we argue that food-animal career development is not reducible to a matter of awareness or recruitment, but a staged process of occupational identity testing through which learners imagine, evaluate, and rehearse possible professional futures.

This study is guided by the PIG Pathway Framework (Table 1), a learner-centered, developmental model for understanding staged occupational identity testing in food-animal veterinary and agricultural education. Rather than treating experiential exposure as a uniform intervention, the framework theorizes how different kinds of experiences do different forms of identity work across educational stages. The framework integrates three complementary theories: Possible Selves Theory, which explains how individuals imagine and evaluate future versions of themselves (13); Social Cognitive Career Theory (SCCT), which emphasizes self-efficacy, outcome expectations, supports and barriers, goal-setting, and career transitions (14); and Experiential Learning Theory, which highlights learning through doing, reflecting, and applying knowledge in context (15). Together, these theories help explain how learners move from imagining a career as possible, to appraising whether it fits, to rehearsing and consolidating a professional identity through mentored participation.

Possible Selves Theory proposes that individuals hold representations of the people they might become, hope to become, or fear becoming (13). These future-oriented representations connect present identity with motivation by making particular goals and consequences personally meaningful. Educational exposure may therefore expand learners’ perceived possibilities by making unfamiliar careers imaginable, while also narrowing or “pruning” possible selves when a career appears inconsistent with their interests, values, abilities, or desired lives.

Social Cognitive Career Theory explains career development through interactions among learning experiences, personal characteristics, behavior, and environmental conditions (14). Learning experiences shape self-efficacy beliefs, i.e., judgments about one’s ability to perform relevant tasks, and outcome expectations, i.e., beliefs about the likely consequences of pursuing a career. These beliefs influence interests, goals, choices, and persistence, while contextual supports and barriers affect whether interests are translated into action. In Pork Prospect, SCCT helps interpret how hands-on practice, mentorship, professional networks, financial considerations, lifestyle expectations, and perceived belonging informed participants’ appraisal of swine-related careers.

The PIG Pathway specifies how established constructs become differently salient across developmental moments. At the Exploratory stage, experiences primarily expand and prune possible selves. At the Formative stage, experiences support appraisal of fit, barriers, self-efficacy, and occupational expectations. At the Immersive stage, experiences enable learners to rehearse a professional self within real or near-real communities of practice.

At the Exploratory stage, high school learners in SDD-Y may have limited familiarity with swine careers but are open to imagining new futures as they approach transitions into higher education or the workforce. Here, Possible Selves Theory explains how exposure can expand their sense of what is achievable, while early affective experiences, such as encouragement or role modeling, begin to shape emerging self-efficacy. Experiential learning is introduced through direct farm exposure that sparks curiosity and makes abstract careers tangible. At this stage, the key educational function of exposure is not commitment, but possibility expansion: learners begin to see food-animal work as a thinkable future, while also identifying paths that may not fit their interests or self-understandings.

At the Formative stage, college students in SDD-C have declared interest in veterinary or animal science but are still exploring specialization. SCCT becomes more central as students evaluate their fit with food-animal careers, including personal values, barriers, and opportunities. Possible Selves are revisited in light of new experiences and encounters with specialists presenting at SDD-C, and Experiential Learning deepens as students reflect more critically on farm experiences, integrating academic knowledge with firsthand exposure and beginning to align personal values with emerging career interests. The Formative stage therefore centers on fit appraisal: learners use structured encounters with animals, specialists, and industry settings to evaluate not only whether they are interested in swine careers, but whether such careers appear livable, attainable, and compatible with their desired professional identities.

At the Immersive stage, Swine Bootcamp participants move beyond exploration and evaluation; they test and enact a potential professional identity through longer, interest-aligned placements. Kolb’s full learning cycle -- experience, reflection, conceptualization, and application -- is most active here, as participants experience, reflect, apply knowledge, and plan next steps. SCCT helps explain how mentorship, self-efficacy, and perceived supports shape persistence, while Possible Selves narrow into concrete professional goals, often articulated in terms of specific roles such as swine veterinarian, farm manager, nutritionist, or producer. In this stage, experiential learning functions as professional rehearsal: participants temporarily inhabit the roles, relationships, routines, and constraints of swine-related work, allowing them to validate, refine, or reconsider their emerging occupational identities.

As such, the PIG Pathway reframes food-animal workforce development as a socially situated process of professional becoming rather than a linear pipeline from exposure to career choice. The framework directs attention to how learners’ career possibilities are shaped not only by knowledge gains, but also by embodied encounters with animals, mentorship, perceived belonging, occupational cultures, and institutional access to professional networks. This framing is especially relevant for veterinary education because decisions to enter food-animal fields are made within social and structural contexts that include financial considerations, such as educational debt and anticipated compensation; lifestyle expectations, including working hours and geographic mobility; gendered and racialized belonging; rural and geographic constraints; and perceptions of professional sustainability.

## 3 Methodology

### 3.1 Study design

This study employed a qualitatively driven convergent parallel mixed methods design, incorporating a one-group pretest-posttest structure for the quantitative strand to examine self-reported changes associated with program participation (see Figure 1) (16–18). The convergent approach allowed for the simultaneous collection and later integration of numeric and textual data, with qualitative findings taking interpretive priority. The intervention, Pork Prospect, comprised three distinct program components aligned with three developmental stages in the PIG Pathway Framework. These components did not constitute a longitudinal sequence completed by the same individuals; instead, they involved separate participant groups recruited at different educational and career stages. Participation in each component was voluntary. These components formed the basis of the intervention under study:

**Figure 1:**
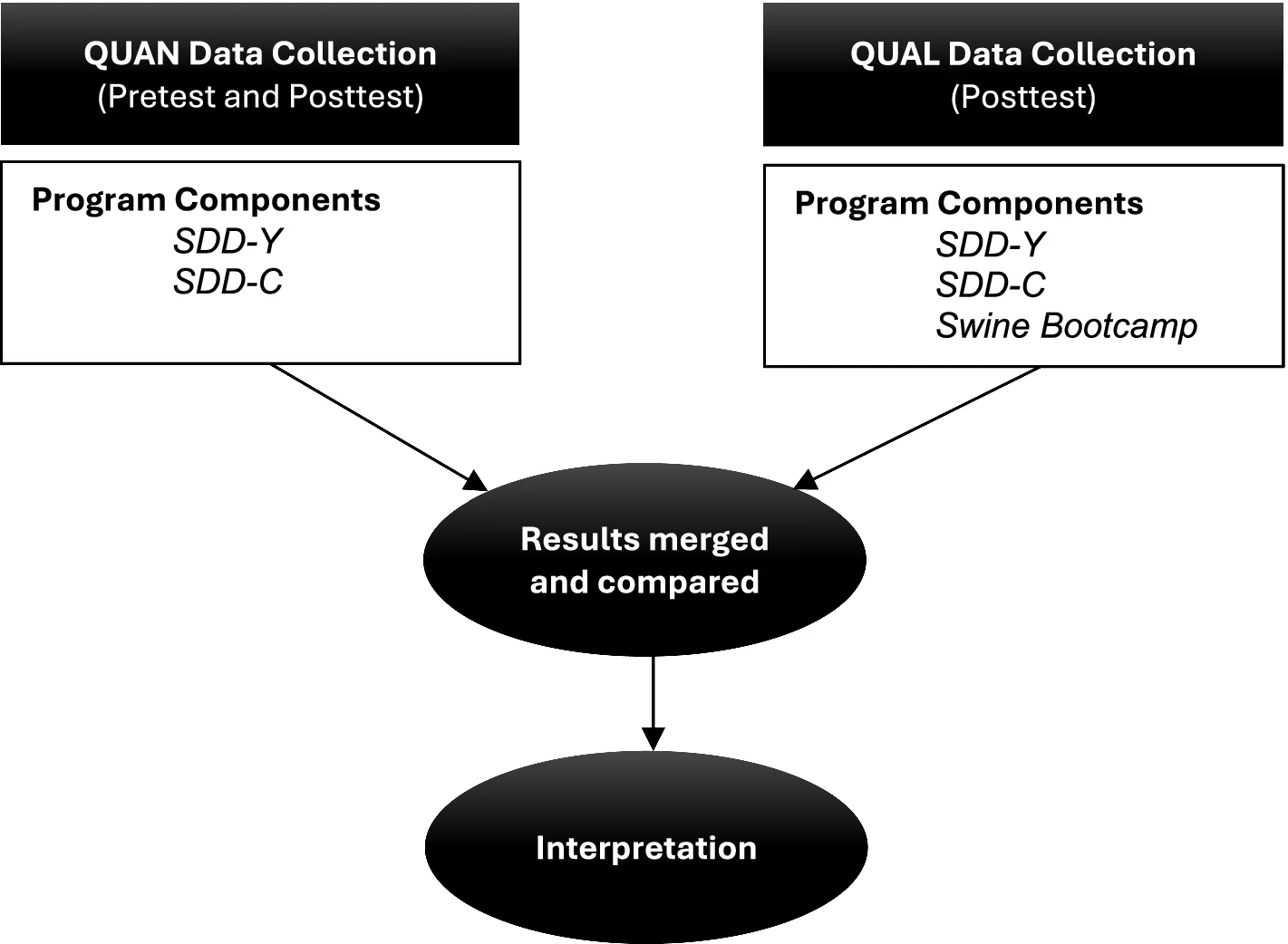
A qualitatively-driven convergent parallel mixed methods design with a one-group pretest-posttest component for studying career development in swine-focused experiential learning programs. Participants in the SDD-Y and SDD-C program components provided both pre-program (pretest) and post-program (post-test) quantitative data (multiple-choice survey questions; QUAN), while Swine Bootcamp participants provided only a posttest. The intervention (program) occurs between the pretest and posttest. Qualitative data (open-ended survey questions; QUAL) were collected only after the intervention (posttest) for all participants. The quantitative and qualitative strands were then merged in the final interpretation.

#### 3.1.1 Swine discovery day – youth and college

Swine Discovery Day-Youth (SDD-Y) and Swine Discovery Day-College (SDD-C) were voluntary, one-day educational events that combined short educational presentations, interactive question-and-answer sessions, guided tours of teaching and research farms, animal handling or diagnostic demonstrations, and supervised hands-on activities. Depending on the host institution, audience, and available speakers, sessions addressed animal welfare, biosecurity, piglet processing, reproduction and artificial insemination, swine medicine, veterinary diagnostics, meat science, nutrition, technology, environmental sustainability, industry economics, and swine-related career opportunities. This broad-based exposure aimed to help students envision diverse career trajectories, or “possible selves,” within the swine industry, grounded in the idea that “what others are now, I could become” (13). While SDD-Y (*n* = 91) examined whether early exposure could increase participants’ awareness of and interest in swine careers, SDD-C (*n* = 58) assessed changes in industry understanding, career awareness, and broadened career consideration among students already enrolled in animal science and veterinary programs.

In SDD-Y, participants completed large animal educational activities before visiting The Ohio State University swine teaching and research farm. At the farm, students participated in a guided tour and rotated through approximately 15-min stations facilitated by academic, veterinary, and industry professionals. Stations incorporated demonstrations, opportunities to interact with materials and animals, and discussion of the associated occupations.

SDD-C events were held at The Ohio State University and the University of Minnesota. Although individual schedules reflected the resources and expertise of each institution, the events generally combined seminars, interaction with professionals, guided farm exposure, animal-handling or diagnostic demonstrations, and applied activities. SDD-C also included guided reflection before and after the farm visit. On the trip to the farm, facilitators invited participants to discuss their previous experiences with large animals, pigs, or other animals; their expectations about encountering pigs; what they hoped to observe and learn; and whether they could envision a swine-related career. On the return trip, facilitators asked participants to reflect on how meeting and working around pigs compared with their expectations, what pleasant or unpleasant aspects of the experience stood out, and whether direct exposure had changed how they imagined a possible career involving swine. These discussions were conversational rather than formally recorded qualitative data and were intended as an educational component of the program.

#### 3.1.2 Swine bootcamp

Swine Bootcamp constituted the Immersive component of Pork Prospect and was designed for advanced undergraduate students, graduate students, and recent graduates seeking in-depth experience in the swine industry. Bootcamp consisted of individualized, professionally mentored experiences lasting from 2 days to 2 weeks. Experiences were tailored to each participant’s interests, goals, availability, and prior experience.

Placements included internships in swine-production settings, work with swine veterinarians, research or diagnostic laboratory visits, extension activities, feed-mill and farm experiences, producer field days, and participation in scientific, policy, or industry events. The research team’s combined relationships with swine-industry, veterinary, academic, extension, and policy actors created a broad network through which participants could be connected with relevant hosts and professional settings.

Each placement incorporated reflection at multiple points. Before the experience, the participant met with a member of the research team to discuss career interests, learning goals, previous experience, and the type of exposure that would be most valuable. During the placement, the team conducted at least one check-in with both the participant and host to evaluate progress, address concerns, and determine whether the experience was meeting the participant’s needs. Afterward, the participant met with the team to reflect on major learning experiences, career fit, unexpected or challenging aspects of the placement, and possible next steps. Participants also completed an open-ended post-program survey, which provided a more private opportunity to document both pleasant and unpleasant experiences.

Because Bootcamp placements occurred at different times and varied substantially in content, reflection was conducted individually rather than through a common focus group. This approach allowed reflection to be tailored to each placement but limited opportunities for participants to share and compare their experiences with those of other Bootcamp participants. In total, 18 participants completed Bootcamp placements.

Each component of the Pork Prospect program operationalized the core components of the PIG Pathway Framework: Possible Selves (P) through exposure to career possibilities and structured reflection; Identity, Motivation, and Self-Regulation (I) through goal-setting, development of self-efficacy, and exploration of perceived supports and barriers; and Grounded Experience (G) through situated, experiential learning. While SDD-Youth (component 1) was delivered once to a single high school cohort, SDD-College (component 2) was offered across five separate cycles to different groups of undergraduate and veterinary students. Swine Bootcamp (component 3) was delivered individually to 18 participants, each of whom engaged in a customized internship aligned with their specific career interests. Table 2 summarizes the total number of cycles and participants across the three components.

**Table 2:** Distribution of study participants and participant recruitment methods across program components.

Pork prospect program component	Program delivery	Number of participants (Total)	Recruitment
SDD-Y	One one-day event	91	Moving from meows to moos
SDD-C	Five one-day events	58	Digital displays Website/link page Campus outreach Classroom announcements Instructor dissemination
Swine Bootcamp	18 individualized placements lasting 2 days to 2 weeks	18	SDD survey expression of interest Direct student inquiries Social media (LinkedIn and Instagram)
Total	N/A	167	–

### 3.2 Data collection and analysis

Participants were recruited primarily from the states of Ohio and Minnesota, two of the top pork-producing states in the U. S. through The Ohio State University and the University of Minnesota (UMN), respectively. Recruitment procedures differed across the three program components, and participation was voluntary in all components.

SDD-Y was incorporated into “Moving from Meows to Moos,” an existing program designed for high school students interested in veterinary medicine and food-animal careers (19).^1^ Recruitment targeted incoming 11th- and 12th-grade students through social media dissemination of a program flyer. Students and their families accessed event information and voluntary registration through a QR code.

SDD-C opportunities were advertised through institutional digital displays, the Ohio Pork Information Center website, a shared link page, campus welcome events, informational tables in animal science buildings, classroom announcements, and communication from animal science instructors. Instructors facilitated dissemination of program information, but participation in SDD-C was voluntary. Some OSU students had opportunities to receive course extra credit for attendance; no extra credit was offered at UMN.

As for Swine Bootcamp participants, those were recruited through three principal routes. First, SDD participants were asked at the end of the post-program survey whether they wished to be considered for Bootcamp. Second, students who independently contacted members of the research team seeking summer or research internship experiences were informed about the program. Third, opportunities were advertised through LinkedIn and other social media channels. Prospective participants met with members of the research team to discuss their interests, career goals, previous experiences, and availability. The team then worked with its network of swine industry, veterinary, research, extension, and policy partners to identify an appropriate experience for each participant. Placement availability and alignment between the participant’s interests and the host organization informed the matching process.

The Bootcamp sample included advanced undergraduate students, graduate students, and recent graduates. Recent graduates were individuals who had completed graduate training and were either entering the job market or actively seeking in-depth experience in a particular area of the swine industry.

#### 3.2.1 Mixed methods integration strategy

Quantitative and qualitative data were collected during overlapping components of program implementation, analyzed independently, and merged during interpretation. This approach enabled the qualitative findings to play a leading explanatory role in contextualizing the descriptive quantitative patterns, yielding a comprehensive understanding of interest in swine careers across learner development stages. Figure 1 offers a conceptual overview of the research design, mapping the structure, timing of data collection in relation to the intervention, and integration of the qualitative and quantitative components. The mixed methods diagram design conventions recommended by Creswell and Plano Clark (18) were followed.

The quantitative strand used a one-group pretest-posttest design. Although pragmatic in educational settings where comparison groups are not feasible, this design does not support causal inference (16, 20). Integrating quantitative and qualitative findings provided greater interpretive depth and enabled comparison across data types.

#### 3.2.2 Quantitative strand

SDD-Y and SDD-C participants completed pre- and post-program Likert-scale surveys administered by QR code. SDD-Y, used a 4-point Likert-scale survey (1: Strongly Disagree, 2: Disagree, 3: Agree, and 4: Strongly Agree), and SDD-C used a 5-point Likert-scale survey (1: Strongly Disagree, 2: Somewhat Disagree, 3: Neither Agree Nor Disagree, 4: Somewhat Agree, 5: Strongly Agree). Question items assessed changes in awareness, comfort, knowledge, and career interest in swine-related fields.

Quantitative analyses were conducted in R (v4.4.0). Survey items were measured on ordinal Likert scales, and preliminary Shapiro–Wilk tests indicated deviations from normality. Therefore, the Wilcoxon signed-rank test was used to assess paired differences between the pre- and post-program responses for each survey statement in the SDD-Youth and SDD-College components. This non-parametric test does not assume a normal distribution and is well suited for evaluating within-subject changes in ordinal data, such as Likert responses. Across all components, means and medians were calculated to provide descriptive summaries of participant attitudes at baseline and following the intervention. The Likert scale from 1 to 4 (SDD-Y) and 1–5 (SDD-C) was used to calculate means and medians for each question item. Statistical significance was assessed at an alpha level of *α* = 0.05.

Swine Bootcamp participants completed post-program surveys only due to the individualized nature and variable timing of participant placements. Surveys were distributed via email after program completion and included similar items assessing the career clarity, confidence, and commitment of participants.

#### 3.2.3 Qualitative strand

Qualitative data were gathered across all three program components using open-ended post-program survey questions. Items were designed to elicit the reflections of participants on program value, their motivations for engagement, their perceived barriers to pursuing swine careers, and their evolving career identities. The analysis combined inductive and deductive thematic coding. Inductive, or emic, coding allowed concepts and patterns to emerge from participants’ responses, whereas deductive, or etic, coding used constructs from Possible Selves Theory, Social Cognitive Career Theory, Experiential Learning Theory, and the PIG Pathway Framework as sensitizing concepts rather than as fixed categories into which every response had to fit (21).

Program implementation and preliminary interpretation initially occurred within each institution. At OSU, Authors 2, 5, and 6 met regularly to plan and implement Pork Prospect and review emerging site-specific observations; Authors 1, 3, and 4 performed the corresponding activities at UMN. The two institutional teams also met twice to coordinate the cross-site research and discuss shared themes and observations. Authors 1 and 2 -- a sociologist of education and health at UMN and a veterinary public health student at OSU, respectively -- subsequently led the integrated qualitative analysis through 22 cross-site analytic meetings. They compared participant responses across sites and program components, examined the findings in relation to the theoretical framework and quantitative results, and refined the themes through discussion and consensus. This structure combined the local contextual knowledge of each institutional team with sustained cross-site interpretation.

The theories underlying the PIG Pathway Framework informed program design, survey development, and interpretation but were not treated as fully operationalized outcome measures. Survey items directly assessed awareness, knowledge, comfort, interest, and career consideration. Constructs such as self-efficacy, outcome expectations, perceived supports and barriers, and future professional identities were used as interpretive lenses when supported by participants’ qualitative responses.

### 3.3 Credibility, trustworthiness, and reflexivity

We approach this work as collaborators brought together by swine, but not by identical relationships to them. Our perspectives span the sociology of education and health, veterinary public health, veterinary medicine, animal science, and extension education. Across these fields, pigs are encountered as living animals, patients, subjects of research, and animals raised within food-production systems. We draw on different knowledge traditions that inform what we consider credible and useful knowledge. These orientations shape what we notice, whom we work with, the questions we ask, and what we understand education to make possible.

Our relationships with students, universities, and veterinary and industry partners enabled Pork Prospect while also shaping which experiences and professional futures we could offer. We therefore understand ourselves as participants in the educational world we are studying, not observers standing outside it. Our commitment to food-animal education brings knowledge and investment, but also expectations about its value. This position calls us to take students’ hesitation, discomfort, and decisions against a swine-related career as seriously as their enthusiasm. We understand informed reconsideration, alongside growing commitment, as a meaningful outcome of career exploration.

Following Olmos-Vega et al. (22), we approached reflexivity as a collaborative examination of how these positions and relationships shaped the research, rather than as an attempt to eliminate subjectivity. During analysis, we considered how our expectations about the program’s value influenced interpretation and used differing perspectives within the team to question program-affirming readings of participants’ accounts.

We addressed qualitative trustworthiness through credibility, dependability, confirmability, and transferability (21). Credibility was supported through collaborative analysis, comparison across participant groups, and integration of quantitative and qualitative findings. Dependability was supported through iterative comparison of developing interpretations with participant responses and transparent reporting of the analytic process. Reflexive examination and the inclusion of both positive and critical participant perspectives supported confirmability. Transferability was supported through descriptions of the institutional settings, participants, recruitment pathways, and program activities, together with participant excerpts illustrating variation across SDD-Y, SDD-C, and Bootcamp. The Pork Prospect Guide provides additional implementation detail.

### 3.4 Ethics statement

This study was approved by the Institutional Review Board at OSU under two exempt determinations: IRB 2024E0427 (for college student participants) and IRB 2022E1030 (for high school student participants). Both protocols were classified as Exempt, Category 1.

## 4 Results

### 4.1 Participant demographics and background

Participants (*N* = 167) were distributed across three developmental components: high school (SDD-Y, *n* = 91), college (SDD-C, *n* = 58), and early career (Swine Bootcamp, *n* = 18). High school participants tended to come from agricultural or rural communities: 35.16% reported living on farms and 32.97% in rural towns. Among SDD-C participants, exposure to farming showed an intergenerational decline: 76.86% reported their grandparents had lived or worked on a farm, compared to 58.62% of parents and just 37.93% of the students themselves. The participants were predominantly female (79.87%) and identified as white (93.96%) across both SDD-Y and SDD-C (Table 3). Among college-level participants, 63.79% were enrolled in animal science programs and 36.21% in veterinary medicine, with most affiliated with either OSU or UMN.

**Table 3:** Demographic characteristics of Swine discovery day- youth (SDD-Y) and swine discovery day–college (SDD-C).

Demographics		Program component		
		SDD-Y (%) *N* = 91	SDD-C (%) *N* = 58	Total (%) *N* = 149
Gender	*Male*	12.09 (11/91)	25.86 (15/58)	17.45 (26/149)
	*Female*	85.71 (78/91)	70.69 (41/58)	79.87 (119/149)
	*Nonbinary/other*	2.2 (2/91)	3.45 (2/58)	2.68 (4/149)
Race	*American Indian or Alaska Native*	1.1 (1/91)	3.45 (2/58)	2.01 (3/149)
	*Asian*	0 (0/91)	10.34 (6/58)	4.03 (6/149)
	*African American*	2.2 (2/91)	1.72 (1/58)	2.01 (3/149)
	*Native Hawaiian/other Pacific Islander*	0 (0/91)	0 (0/58)	0 (0/149)
	*White*	100 (91/91)	84.48 (49/58)	93.96 (140/149)
	*Other/missing*	0 (0/91)	1.72 (1/58)	0.67 (1/149)
Ethnicity	*Non-Hispanic / Non-Latino*	94.51 (86/91)	79.31 (46/58)	88.59 (132/149)
	*Hispanic / Latino*	3.30 (3/91)	13.79 (8/58)	7.38 (11/149)
	*Other/missing*	2.2 (2/91)	3.45 (2/58)	2.68 (4/149)
Educational level	*High School*	100 (91/91)	0 (0/58)	61.07 (91/149)
	*College (Freshman)*	0 (0/91)	20.69 (12/58)	8.05 (12/149)
	*College (Sophomore)*	0 (0/91)	17.24 (10/58)	6.71 (10/149)
	*College (Junior)*	0 (0/91)	24.14 (14/58)	9.39 (14/149)
	*College (Senior)*	0 (0/91)	12.07 (7/58)	4.69 (7/149)
	*Other (Graduate level or early career professionals)*	0 (0/91)	25.86 (15/58)	10.07 (15/149)

### 4.2 Quantitative results

#### 4.2.1 Exploratory stage (SDD-youth)

At the Exploratory stage, we sought primarily to raise the familiarity of high-school students with swine and to spark early career identity exploration. Table 4 summarizes the pre- and post-program ratings, while Figure 2 depicts the distributional shifts for each statement. Before Swine Discovery Day, participants expressed only moderate ease around pigs (mean = 3.36, median = 3.5). After the event, the mean rose to 3.52 and the median to 4, a modest but significant increase (*p* = 0.0196; Figure 2A). A much larger shift occurred in self-reported knowledge of the swine industry: the mean climbed from 2.78 to 3.58 and the median from 3 to 4 (*p* < 0.001; Figure 2B). Awareness of career opportunities followed a similar pattern, increasing from 2.68 to 3.41, (*p* < 0.001; Figure 2C). Although initial interest in pursuing a swine career was low (mean = 2.05, median = 2), the post-program survey still registered a significant uptick to 2.28 (median = 2, *p* = 0.005; Figure 2D). Collectively, results indicate that even a single-day intervention can measurably improve comfort, knowledge, and career awareness among youth who previously had limited exposure to swine production.

**Table 4:** Pre- and post-program means, medians, and Wilcoxon signed-rank test results for SDD-Y participants.

Statement	Pretest		Posttest		*p*-value
	Mean	Median	Mean	Median	
I feel comfortable working around pigs	3.36	3.5	3.52	4	0.0196
I know about the swine industry	2.78	3	3.58	4	< 0.001
I am aware of career opportunities in the swine industry	2.68	3	3.41	3	< 0.001
I am considering a career in the swine industry	2.05	2	2.28	2	0.005

**Figure 2:**
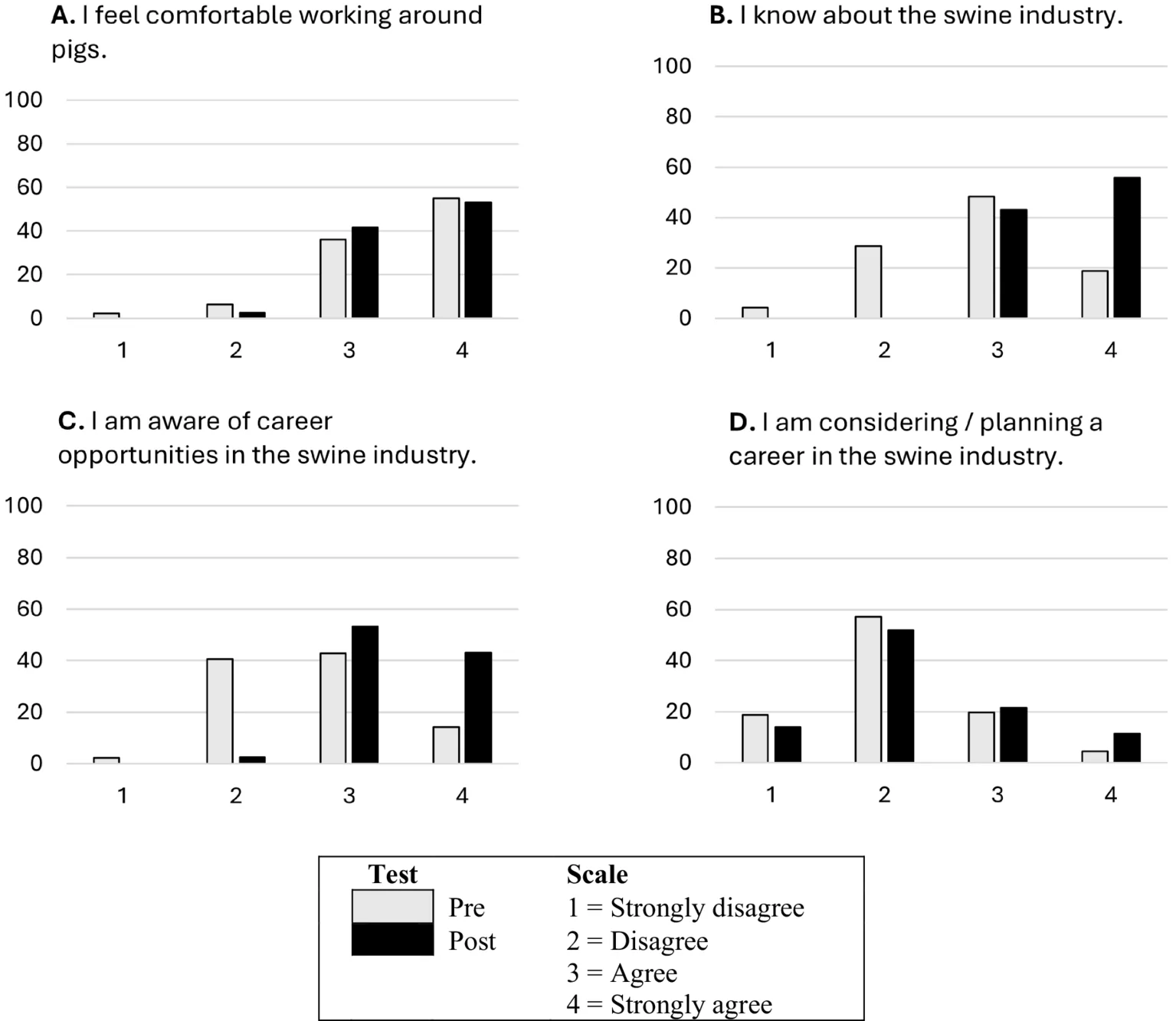
Pre- and post-program (Before and After, respectively) responses to survey statements for high-school participants in Swine Discovery Day (SDD-Youth): **(A)** Comfort working around pigs. **(B)** Knowledge of the swine industry. **(C)** Awareness of career opportunities in the swine industry. **(D)** Consideration of a career in the swine industry. The vertical axis shows respondents (%), and the horizontal axis shows Likert response categories; bars distinguish pre- and post-program responses. All four pre–post comparisons were statistically significant (paired Wilcoxon signed-rank tests, *p* < 0.05); exact *p*-values are reported in Table 4.

#### 4.2.2 Formative stage (SDD-college)

College participants began the Formative stage with declared interests in animal or veterinary sciences but were still refining their professional focus. As shown in Table 5, their baseline understanding of the swine industry was already above neutral (mean = 3.47, median = 4); nevertheless, the post-program survey recorded a significant rise to a mean of 4.92 and a median of 5 (*p* < 0.001; Figure 3C). Awareness of career opportunities displayed a comparable gain, increasing from 3.69 to 4.74 and shifting the median from 4 to 5 (*p* < 0.001; Figure 3A). The most pronounced change occurred in career intent: consideration of a swine-related career advanced from a neutral stance (mean = 3.18, median = 3) to a clearly positive one (mean = 4.26, median = 4; *p* < 0.001; Figure 3B). Results indicate that, for students already engaged in animal-science disciplines, the Formative-stage curriculum not only deepened their technical understanding of the industry but also translated that knowledge into a substantially stronger inclination to pursue swine-focused careers.

**Table 5:** Pre- and post-program changes in swine industry knowledge, career awareness, and interest among SDD-C participants.

Statement	Pretest		Posttest		*p*-value
	Mean	Median	Mean	Median	
I have a good understanding of the swine industry	3.47	4	4.92	5	<0.001
I am aware of career opportunities in the swine industry	3.69	4	4.74	5	<0.001
I am considering a career in the swine industry	3.18	3	4.26	4	<0.001

**Figure 3:**
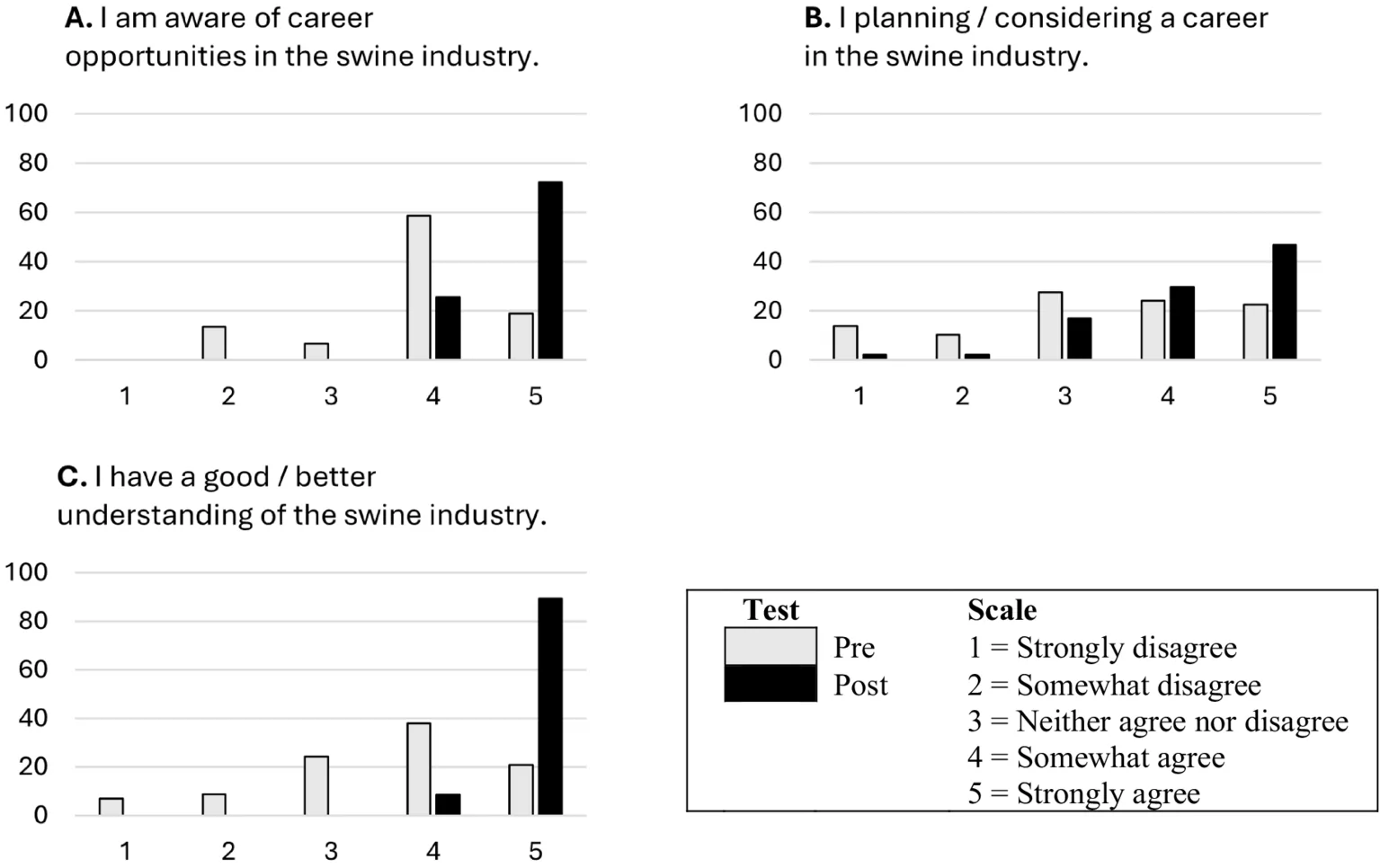
Pre- and post-program responses to survey statements for college participants in Swine Discovery Day (SDD-College): **(A)** Awareness of career opportunities in the swine industry. **(B)** Consideration of a career in the swine industry. **(C)** Understanding of the swine industry. The vertical axis shows respondents (%), and the horizontal axis shows Likert response categories; bars distinguish pre- and post-program responses. All three pre–post comparisons were statistically significant (paired Wilcoxon signed-rank tests, *p* < 0.001).

When asked about what attracted them to the swine sector, the majority of SDD-C participants (87.93%) cited a strong intrinsic interest in agricultural sciences and food animals (Figure 4). Career-related benefits such as income potential and growth opportunities were the next most common motivator, selected by 63.79% of respondents. Other pull factors included environmental stewardship (27.59%), family tradition and interest in agricultural technologies (both at 20.69%), and entrepreneurial aspirations (10.34%).

**Figure 4:**
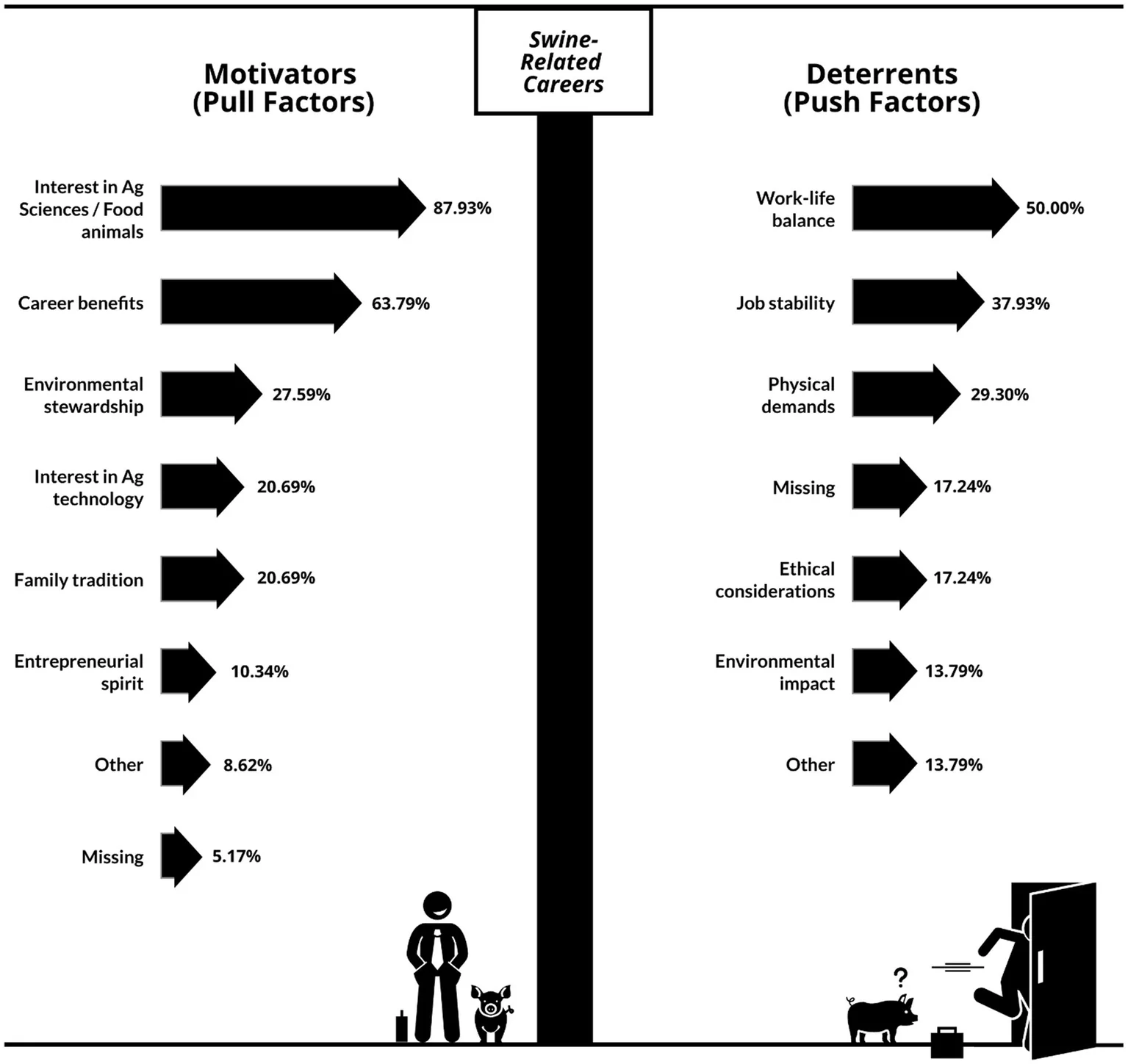
Reported motivating (“pull”) and deterring (“push”) factors concerning swine-related careers among SDD-C participants. Inward-pointing arrows indicate motivating factors, and outward-pointing arrows indicate deterring factors; percentage labels show the reported frequency of each category. Entries labelled “Missing” represent item nonresponse, not motivating or deterring factors, and are displayed only to document missing data. Arrow size corresponds to the percentage of respondents citing each factor. Figure generated using Piktochart software.

At the same time, students expressed reservations about entering the swine industry. The most commonly reported push factor was concern about work-life balance (Figure 4), reported by 50% of respondents, followed by job stability (37.93%) and the physical demands of work (29.31%). Fewer participants pointed to environmental or ethical concerns (13.79 and 17.24%, respectively), while some cited additional deterrents such as geographic barriers, a lack of prior experience, and perceived exclusivity within the industry, as one participant noted that the swine industry “might not always be a welcoming industry toward women and foreigners”.

Additionally, 77.59% of participants reported feeling comfortable working with swine, while 12.06% were indifferent and 10.35% expressed discomfort. Enthusiasm for the program was high: all respondents (*n* = 46) agreed or strongly agreed that they would recommend Pork Prospect to others. Regarding future engagement, 38.3% expressed interest in participating in Swine Bootcamp, 51.1% were uncertain, and 10.6% were not interested.

#### 4.2.3 Immersive stage: swine bootcamp

Swine Bootcamp participants were advanced undergraduates or early-career professionals in animal science or veterinary fields who entered the program with clearly defined interests in food-animal systems. Unlike earlier program components, Bootcamp placements were tailored to each participant’s career focus, making this a highly individualized, immersive experience.

Post-program survey responses (Figure 5) indicated that all Swine Bootcamp participants felt the experience improved their knowledge of swine production and career pathways, and all expressed willingness to recommend the program to others. Additionally, 72.22% of participants agreed or strongly agreed that Bootcamp increased their interest in pursuing a swine-related career, while 22.22% were neutral and one participant (5.56%) disagreed.

**Figure 5:**
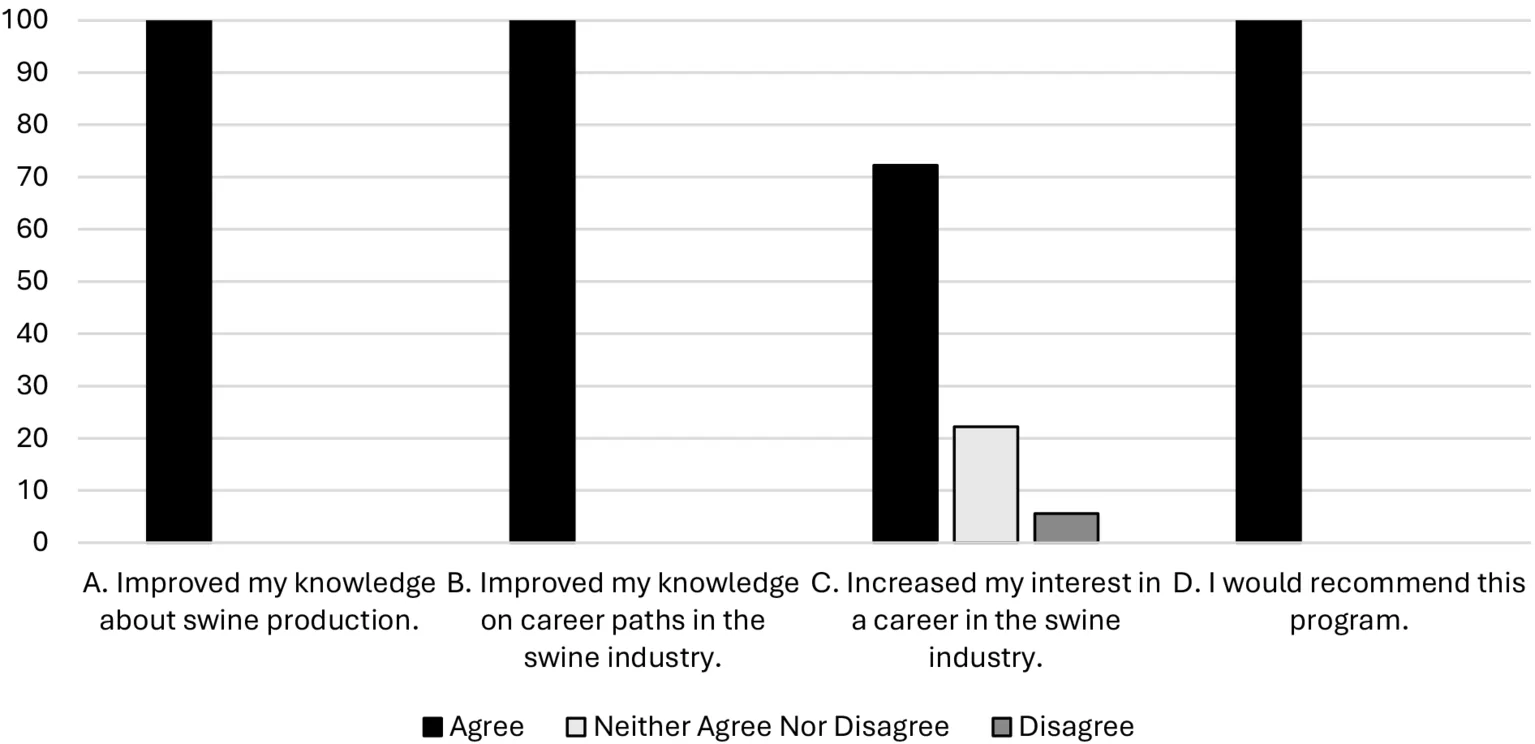
Post-program agreement of Swine Bootcamp participants (*n* = 18) with statements about program impact. The vertical axis shows respondents (%); grouped bars represent the agreement categories identified in the legend.

### 4.3 Qualitative results

#### 4.3.1 Exploratory stage (SDD-youth)

The feedback collected from high school students participating in SDD-Y reveals a range of insights into their experiences, preferences, and future aspirations. SDD-Y participants articulated their possible selves through statements about species preferences, desired universities, or, notably, in some cases, a clear realization that they did *not* want to become veterinarians:

*[…] I do not plan on being a vet, [but] I enjoyed the experience.* (SDD-Y Participant 9).

*[SDD-Y] was a lot of fun, it helped me see more opportunities for myself in the future.* (SDD-Y Participant 12).

These excerpts suggest that exploration of possible selves includes not only aspirational identities, but also the active pruning of less desirable futures that help students narrow and refine their sense of vocational fit.

While the program intended to focus on swine, an unintended consequence of that focus was students identifying interest in other species (beyond swine), which is also a selective process where students further refine their professional aspirations and align them with their passions:

*I [wanted to do more] with smaller animals and livestock animals other than horses, cows, and swine. I would like to see more sheep, poultry, rabbits, etc.* (SDD-Y Participant 5).

*I thought it was pretty informative. I would have loved to see more animals such as the sheep and goats just to get a good idea of how they are cared for on the farm […].* (SDD-Y Participant 10).

A few students (*n* = 3) explicitly highlighted the value of hands-on learning, which was a key feature of the program. Phrases like “I […] love the hands on!!” (SDD-Y, Participant 13) and specific references to activities such as working with swine organs, laboratory micropipettes, and hand ties suggest that the experiential nature of the program enhanced students’ understanding and interest in veterinary medicine.

#### 4.3.2 Formative stage (SDD-college)

Although only a few high school students (*n* = 3) explicitly mentioned their appreciation of hands-on learning when reflecting on their experience, this dimension emerged as central during the Formative stage of engagement. When asked which sessions they enjoyed most, SDD-C participants (*n* = 25) highlighted those involving direct, embodied interaction -- particularly visits to swine facilities -- while sessions focused on financial or business topics were consistently described as least engaging.

Across the SDD-C open-ended responses, participants consistently highlighted their interactions with small pigs and the nursery area as the most memorable part of the program, using phrases such as “Holding the baby piglets” and “Touring the nursery” (SDD-C Participant 30 and 33, respectively). When asked about their least favorite parts of the program, SDD-C participants reported moments of physical discomfort or confrontation with the more instrumental and technical aspects of animal handling such as “being in the pig pen” (SDD-C Participant 23), “getting bitten” (SDD-C Participant 27), “drawing blood from piglets” (SDD-C Participant 32), and “snaring” (SDD-C Participant 33).

Like SDD-Y participants, SDD-C participants also reflected on the kinds of careers they were in:

*[…] Prior to today, I was not considering veterinary medicine as it pertains to industry animals. Despite this, I am much more interested in learning about the potential opportunities that might exist in those types of fields! I do not think my mind would’ve been so open unless I had attended today!!* (SDD-C Participant 15).

*This was super informative and definitely a positive experience that made me more likely to consider working in the swine industry in the future.* (SDD-C Participant 4).

#### 4.3.3 Immersive stage: swine bootcamp

Swine Bootcamp participants (*n* = 6) appreciated their mentors from each unique experience, with some participants either naming specific people that left an impression, or identifying specific roles that:

*I enjoyed getting to know industry professionals, especially [Tommy] and learning about his personal career path. I enjoyed how unique his perspective was as he had worked in lots of different positions within the swine industry. [Tommy] had a diverse background which made the finisher farm visit extra informative!* (Swine Bootcamp Participant 2).

*[…] I loved being able to be around swine vets and learn from them as well as the other workers present. My favorite activity was farrowing. I am really glad I was able to go on this trip and make connections.* (Swine Bootcamp Participant 4).

*[I liked] [the] chance to spend time with on-farm employees and managers and their firsthand expertise.* (Swine Bootcamp Participant 7).

*Loved getting to see a larger-scale farm and hearing directly from veterinarians about swine care during the barn tour.* (Swine Bootcamp Participant 12).

*[I liked] [the] opportunity to feel like a swine vet for a day.* (Swine Bootcamp Participant 14).

It is worth noting that not all participant experiences were positive, and the most frequently reported dissatisfactions clustered around logistical planning (*n* = 5), aspects of the farm environment (*n* = 4), and the scope of the content (*n* = 3).

A few comments described discomfort during the farm visit (*n* = 4). Students referenced strong odors, uncertainty around biosecurity procedures, and limited availability of undergarments needed for protective gear as aspects that made this portion of the experience less appealing.

*A graphic/poster about bio security practices near the Danish entry or shower room [would have helped].* (Swine Bootcamp Participant 12).

A smaller set of responses (*n* = 3) pointed to the scope and relevance of the content itself. These participants wished the bootcamp experience included activities more closely aligned with their specific interests, such as meat processing or additional laboratory and research exposure.

Finally, Bootcamp participants described seemingly clearer career intentions. Their reflections pointed to emerging goals that spanned cross-disciplinary interests, geographic ambitions, and early specialization within veterinary medicine:

*[…] Moving forward, I would be interested in complementing [the Bootcamp] experience by shadowing or interning in policy settings with industry stakeholders to deepen my understanding of the social and policy dimensions beyond the academic space.* (Swine Bootcamp Participant 6).

*Certainly Summer 2024 made me very resilient in understanding how to think on my feet from ensuring that I had the resources to travel around the Midwest to get the opportunity to work with big names in the swine industry. My experience was an awesome opportunity to engage with different types of management of farms and to work close hand with teams in [UMN] CVM who are researching and looking to understand future significant health [concerns] in the swine field not only in the Midwest and the US, but also internationally.* (Swine Bootcamp Participant 9).

*Thank you so much for such an educational opportunity. I have a better idea of what branch of veterinary medicine I want to go into now.* (Swine Bootcamp Participant 17).

## 5 Discussion and conclusions

### 5.1 Possible selves

High school (SDD-Y) participants began with broad, exploratory visions of who they might become. Some discovered new interests, while others realized certain paths were not for them. Such aspirational and aversive reflections indicate that even one-day exposures can influence the self-concept of young learners by expanding or narrowing their perceived career options. This aligns with Possible Selves Theory, which holds that meaningful exposure can prompt youth to imagine -- and also eliminate -- roles that feel (un)suitable (13).

By the college component (SDD-C), Possible Selves became more focused and grounded in the ongoing training of participants. These students, who had already declared majors in animal science or veterinary medicine, used the program to explore specialization within the food-animal domain. Our quantitative results showed a dramatic increase in consideration of swine-related careers after the SDD-C event. Qualitative findings echoed this shift: no SDD-C respondent expressed outright disinterest in food-animal careers; instead, many expressed a positive program experience. This finding reinforces the idea that early-career learners benefit from seeing clear examples of specialists in action. The swine-specific experiences of Pork Prospect likely contributed to such realignments by giving students tangible role models and scenarios to envision themselves in. Consistent with our results, one study found that introducing first-year veterinary students to swine through lectures and farm visits also significantly improved their swine knowledge and attitudes (9).

By the immersive component (Swine Bootcamp), Possible Selves of participants had crystallized into professional goals. The advanced undergraduates or (post)-graduates entered Bootcamp already leaning toward swine or food-animal careers; the intensive, hands-on placements then helped confirm and sharpen their aspirations. Participants spoke in terms of specific roles and future plans; for instance, imagining themselves as swine veterinarians, nutritionists, or farm managers, and even identifying niche interests like policy advocacy or international swine health. An important insight from the Swine Bootcamp is the extent to which the imagined futures of participants were mediated by the institutional relationships embedded in Pork Prospect. The placements the research team was able to offer were not neutral; they were made possible through the existing partnerships of the PIs and co-PIs. This inevitably shaped the horizon of what participants could see for themselves. The participant who asked for exposure to meat processing makes this particularly visible, reminding us that the terrain of “Possible Selves” is never truly open. Rather, it is structured by access, networks, and the opportunities institutions are positioned to provide.

The investigators’ involvement in designing and delivering Pork Prospect provided valuable contextual knowledge but may also have favored program-affirming interpretations. In addition, the team’s professional networks enabled individualized placements while shaping the range of occupational settings and career possibilities available to participants. Collaborative cross-site analysis and attention to critical and disconfirming responses helped address, but could not eliminate, these influences. Future evaluations could include qualitative analysts who were not involved in program delivery.

### 5.2 Identity, motivation, and self-regulation

The Identity, Motivation, and Self-Regulation component of the PIG Pathway (grounded in SCCT) became increasingly salient at the formative and immersive stages, as students moved closer to actual career decisions. In the high school cohort, identity-related motivations were still in formation, meaning participants were simply curious or becoming generally interested in animals. Quantitatively, SDD-Y teens began with low intent toward swine careers, but even here, a small yet significant post-program increase was observed. From a career development view, this is appropriate for their stage because they are gaining exposure and starting to form an initial sense of fit without yet having firm convictions. Prior work on youth agricultural education supports this -- early positive encounters (such as 4-H or FFA activities) build a sense of belonging and self-efficacy in agricultural domains (6, 7), which lays the groundwork for later identity development. Our SDD-Youth participants’ feedback that the event “helped me see more opportunities for myself in the future” (SDD-Y Participant 12) aligns with that mechanism of broadening horizons.

By contrast, college students in SDD-C voiced more differentiated motivations and concerns, indicating they were actively negotiating their emerging professional identity. Before the program, many in this group already had moderate familiarity with the swine industry, and a majority reported a genuine interest in food animals as a major pull toward the field. After the program, not only did their factual understanding of swine production surge, but their expressed willingness to consider a swine-related career jumped from a neutral stance to a decisively positive one. In line with SCCT, we interpret this as a boost in self-efficacy and outcome expectations; through the event’s hands-on sessions and networking, students likely felt more capable of working with swine and more optimistic about the rewards of doing so. Indeed, qualitative remarks made by participants matched that confidence and comfort with swine after the farm tours and practice activities. Our data also indicate that students were weighing perceived supports and barriers during this stage. When asked what might deter them from a swine career, half cited work-life balance concerns, and sizable proportions mentioned job stability and the physical demands of swine practice. These practical considerations echo findings in the broader veterinary literature: for example, surveys of vet students have long shown that lifestyle and workload are key factors steering graduates away from large-animal fields (e.g., concerns about long hours and limited flexibility) (10, 11). This pattern also aligns with agricultural career-intention research showing that the movement of students toward agricultural careers is shaped by interest, perceived fit, social influences, and perceived barriers (23).

Our findings also resonate with Payne et al. (3), who found that women, students from marginalised ethnic groups, and those from urban or suburban backgrounds reported feeling less able to pursue farm practice. Participants’ concerns about whether the swine industry would welcome women and foreigners similarly locate occupational fit within social relationships and professional cultures, rather than individual interest alone. This comparison suggests that experiential programs should address belonging and access alongside technical exposure.

At the Swine Bootcamp stage, we saw participants actively exercising self-regulation and goal-setting as developing professionals. The individuals self-selected into an intensive experience to experiment with what would be their career choice, demonstrating a high level of intrinsic motivation and initiative by committing to the Bootcamp. All Bootcamp participants agreed that the experience improved their knowledge and nearly three quarters said it further increased their interest in a swine-related career. Qualitative feedback illustrates how they used the immersive component to solidify their identities: for example, one participant planned to supplement their hands-on farm knowledge with policy internship experiences, recognizing a multidisciplinary path for their future self. Another described traveling across the Midwest to work with leading swine veterinarians and research teams, reflecting deliberate effort to build networks and skills aligned with their career goals. These planned behaviors correspond to the self-regulatory processes described in SCCT and identity development theories, whereby by seeking out experiences, reflecting on them, and adjusting future plans accordingly, learners are actively constructing their professional identity. It is noteworthy that Bootcamp participants frequently mentioned mentorship and professional networking as highlights. They valued getting to know industry professionals and feeling treated as colleagues or “swine vets for a day.” Such interactions likely boosted their confidence and commitment by providing social support and validation, which are key social cognitive factors that sustain motivation. This outcome is in line with earlier calls in the veterinary education literature to improve mentorship and real-world exposure for food-animal students (11).

In a different practice context, Nappier et al. (24) found that veterinary students’ positive perceptions of and interest in companion-animal primary care increased over an academic year. The authors proposed greater opportunities for positive interactions with primary-care faculty as a possible explanation. Although that mechanism was not directly tested, it offers a useful parallel to the importance participants attributed to mentorship and professional relationships in Pork Prospect.

### 5.3 Grounded experience

Consistent with Experiential Learning Theory (15), the most consequential learning moments in Pork Prospect were often embodied rather than simply informational. Across components, participants learned about swine careers through lectures and presentations, but more importantly, while also encountering the animals, sensory spaces, tools, routines, and social relations through which swine work becomes meaningful. High school and college students repeatedly described handling piglets and touring nursery spaces as memorable and enjoyable, suggesting that emotionally positive animal interactions helped make swine work feel more approachable.

At the same time, participants also confronted the less glamorous realities of swine production during the program, and these encounters were pivotal learning experiences. College students mentioned aspects like drawing blood, restraining adult pigs, being bitten, or simply enduring the strong farm odors as their least favorite moments. Several Bootcamp participants likewise commented on the physical and sensory discomforts – for example, adjusting to biosecurity protocols (and awkward farm attire), or the smell in large finishing barns. These reactions are important because they represent the move from concrete experience to reflective observation. When confronted with a challenging or uncomfortable task, students are prompted to reflect on whether they can or are willing to handle this in the long term. Some participants, despite initial discomfort, came away with increased confidence; for example, one Bootcamper noted that spending time with a veterinarian made them more certain that they could thrive in that role. Others recognized their limits or dislikes, which is equally valuable information for career choice. This balance of attraction and discomfort is echoed in other studies of veterinary student experiences. For instance, Gonzalez et al. (10) described how elective pre-veterinary courses can both inspire students and surface their reservations, allowing them to make informed decisions about where they fit in the profession. Our findings extend this discussion by highlighting the emotional and sensory dimensions of career exploration: the same aspects of swine work can attract some participants while prompting others to reconsider their fit. Thus, effective experiential learning in agriculture must actively navigate this tension, and guided reflection is essential to that process. For example, Ventura et al. (9) observed that what farm tour facilitators emphasize during a visit can shape how students interpret and remember swine management practices. Within Pork Prospect, we sought to scaffold reflection before, during, and after the farm visit. On the drive to the site, the lead veterinarian invited students to situate themselves, which helped surface assumptions and set expectations. During the visit, the farm manager and veterinarian narrated the production spaces, directing attention to particular practices and ways of seeing the farm. On the return trip, we facilitated informal debriefs that captured the first impressions of students, which often sedimented into longer-term understandings of food-animal work. Moving forward, deliberately incorporating more structured reflection (e.g., guided discussions about ethical challenges or lifestyle implications) could further enhance the impact of Pork Prospect.

### 5.4 Implications for theory, practice, and future research

The integrated quantitative and qualitative findings illustrate how a developmentally staged, experiential program can influence students’ interest in food-animal careers. SDD-Youth broadened awareness and planted seeds of curiosity, SDD-College deepened understanding and began aligning interests with career possibilities, and Swine Bootcamp supported commitment through immersive practice. The PIG Pathway Framework helps interpret these shifts as part of a broader process of career formation, in which learners imagine who they might become, assess where they might belong, and test what kinds of professional lives feel possible. Exposure, in this sense, is informational, affective, relational, and embodied, shaped by the people, animals, institutions, and occupational worlds learners are invited to enter.

The sequential logic of the PIG Pathway Framework met students where they are developmentally by providing the right kind of exposure at the right time. At the Exploratory stage, experience opened space for possibility. Students encountered swine work as a world they may not have previously known how to imagine, and in doing so, began to name futures that felt appealing, unfamiliar, or unsuitable. At the Formative stage, experience became a site of appraisal. Learners weighed their interest in swine work alongside questions of competence, lifestyle, belonging, physical demands, and anticipated barriers. At the Immersive stage, experience became a form of rehearsal. Participants moved through the routines, relationships, constraints, and responsibilities of swine-related work in ways that allowed them to refine, deepen, or reconsider their emerging professional identities.

This staged reading deepens the use of Possible Selves Theory, Social Cognitive Career Theory, and Experiential Learning Theory in the context of food-animal veterinary education. Possible selves take shape through concrete access to species, people, places, and professional roles. Self-efficacy and outcome expectations develop through situated encounters with mentors, animals, farm environments, biosecurity practices, and professional networks. Experiential learning carries particular weight in food-animal contexts because learners are asked to engage the sensory, ethical, physical, geographic, and relational realities of the work. The PIG Pathway therefore frames food-animal workforce development as a process of species-situated professional becoming, in which learners come to understand both the field and themselves through participation.

For the veterinary profession, these findings suggest that recruitment into food-animal practice should not rely on information alone. Learners also need opportunities to interact with animals and professionals, assess the sensory and lifestyle realities of the work, develop supportive professional relationships, and determine whether food-animal practice is compatible with their abilities, values, and desired lives. Developmentally sequenced experiences may therefore complement veterinary curricula by supporting informed specialization decisions and connecting interested learners with areas of documented workforce need.

While not all institutions may be able to implement a three-tiered program, our results suggest that even brief interventions can have meaningful effects. For example, a single day program like SDD-C improved students’ swine industry knowledge and produced a positive shift in career consideration. This highlights an opportunity for scalable curriculum enhancements whereby veterinary and animal science programs might integrate joint one-day intensive modules or workshops (even if a full elective course is not feasible) to spark or reinforce interest in food-animal fields. The Pork Prospect Guide was developed to support institutions interested in replicating the program or selected components of it (25).^2^ Such additions could help expand student awareness of livestock-related career pathways and contribute to workforce development across the animal agriculture sector.

Pork Prospect was designed around swine, but the developmental logic of the PIG Pathway can inform programs in other food-animal fields. The framework invites educators to ask what learners need at different moments in their formation: what helps them imagine a species-specific career, what helps them evaluate whether that career fits, and what helps them rehearse the work with enough depth to make informed commitments. Educators and researchers working in poultry, dairy, bison, gamebird, small-ruminant, aquaculture, or other food-animal contexts could use this logic while adapting the species-specific content, technical skills, welfare questions, biosecurity practices, sensory environments, career roles, and mentorship networks.

Finally, a natural next step is to investigate the long-term impact, generalizability, and implementation of the Pork Prospect program across institutional contexts. This study was cross-sectional in the sense that different groups participated in different components; future research should adopt a longitudinal design to track the same individuals as they progress from exploratory to immersive experiences. For instance, it would be informative to follow high school SDD-Y participants into college to determine whether early exposure increases their likelihood of enrolling in animal science programs or attending events like SDD-C down the line. Similarly, following SDD-C participants into their postgraduate choices would provide direct evidence of conversion from interest to entry. Qualitative follow-up interviews could also shed light on which program elements had the most lasting influence on participants’ career decisions.

In addition, future research should examine how the program is adapted and implemented across different universities, including which components are retained, modified, or expanded in response to the focal species of interest, local resources, student populations, and industry partnerships. Such comparisons could assess variation across settings and inform decisions about institutional investment in these programs. Adaptation across species should be studied as a substantive question in its own right. Different food-animal fields organize access, labor, expertise, ethical concern, animal-human relations, and professional belonging in distinct ways. Expanding the program to new sites and more diverse populations is also important. Our participant pool, while sizable (*N* = 167), was largely homogenous in terms of race/ethnicity and tied to two land-grant universities in two major pork-producing states. Engaging students from underrepresented backgrounds and different regions will be crucial for broadening the pipeline of food-animal professionals – an aim that aligns with recent recommendations to improve inclusivity in veterinary education (3). Future research should therefore examine how programs like Pork Prospect shape whose futures become visible, credible, and supported within food-animal veterinary education, and how institutions might widen access to those futures.

## Data Availability

The materials supporting the findings of this study are included in the article, Supplementary Material, and publicly available program materials cited in the manuscript. Participant-level data are not publicly available due to confidentiality and re-identification concerns, particularly for qualitative responses containing contextual information about participants, institutions, and industry partners. Requests for de-identified quantitative data or additional analytic materials may be directed to the corresponding author and will be considered subject to institutional, ethical, and confidentiality requirements.

## Footnotes

1 Blinded for peer review.

2 Blinded for peer review.
